# Measured and perceived environmental characteristics are related to accelerometer defined physical activity in older adults

**DOI:** 10.1186/1479-5868-9-40

**Published:** 2012-04-03

**Authors:** Scott J Strath, Michael J Greenwald, Raymond Isaacs, Teresa L Hart, Elizabeth K Lenz, Christopher J Dondzila, Ann M Swartz

**Affiliations:** 1 Department of Kinesiology, University of Wisconsin-Milwaukee, Milwaukee, WI, USA; 2 Urban Design 4 Health, Inc., Seattle, WA, USA. Current: Lane Council of Governments, Eugene, OR, USA; 3 School of Architecture and Urban Planning, University of Wisconsin-Milwaukee, Milwaukee, WI, USA; 4 The College at Brockport, State University of New York, Brockport, NY, USA

## Abstract

**Background:**

Few studies have investigated both the self-perceived and measured environment with objectively determined physical activity in older adults. Accordingly, the aim of this study was to examine measured and perceived environmental associations with physical activity of older adults residing across different neighborhood types.

**Methods:**

One-hundred and forty-eight older individuals, mean age 64.3 ± 8.4, were randomly recruited from one of four neighborhoods that were pre-determined as either having high- or low walkable characteristics. Individual residences were geocoded and 200 m network buffers established. Both objective environment audit, and self-perceived environmental measures were collected, in conjunction with accelerometer derived physical activity behavior. Using both perceived and objective environment data, analysis consisted of a macro-level comparison of physical activity levels across neighborhood, and a micro-level analysis of individual environmental predictors of physical activity levels.

**Results:**

Individuals residing in high-walkable neighborhoods on average engaged in 11 min of moderate to vigorous physical activity per day more than individuals residing in low-walkable neighborhoods. Both measured access to non-residential destinations (b = .11, *p *< .001) and self-perceived access to non-residential uses (b = 2.89, *p *= .031) were significant predictors of time spent in moderate to vigorous physical activity. Other environmental variables significantly predicting components of physical activity behavior included presence of measured neighborhood crime signage (b = .4785, *p *= .031), measured street safety (b = 26.8, *p *= .006), and perceived neighborhood satisfaction (b = .5.8, *p *= .003).

**Conclusions:**

Older adult residents who live in high-walkable neighborhoods, who have easy and close access to nonresidential destinations, have lower social dysfunction pertinent to crime, and generally perceive the neighborhood to a higher overall satisfaction are likely to engage in higher levels of physical activity behavior. Efforts aimed at promoting more walkable neighborhoods could influence activity levels in older adults.

## Background

Regular physical activity has been shown to reduce coronary heart disease, type 2 diabetes mellitus, obesity, and some forms of cancer [1]. Physical activity has also been shown to improve sleep quality [2], prevent or delay cognitive impairment [3], and improve physical function [4]. Despite the importance of physical activity in preserving health, physical activity levels decline with advancing age, whereby only 30-35% percent of the U.S. population over the age of 65 years are engaging in minimum amounts of physical activity necessary to confer health benefits [5]. Aging statistics show that people over the age of 65 are the fastest growing segment of the U.S. population [6], and that 1 in 2 older adults have a chronic disease or disability [7]. Therefore, efforts to promote increased levels of physical activity have become a national health objective.

Increasing or maintaining regular physical activity incorporates a complex interaction between the individual and the environment, and because of such, public health advocates have expanded their overall conceptualization of physical activity behavior to incorporate not only individual variables (self-efficacy, beliefs, intentions etc.) but also social, physical, organizational and political environments [8]. Studies show, based upon selected environmental characteristics, namely density, connectivity and land-use mix, that neighborhoods can be deemed as either more- or less-walkable and be strongly linked to physical activity levels [9,10]. Of late, there has been an emergence of evidence linking numerous components of the neighborhood environment to physical activity behavior, and excellent reviews exist on this topic [11,12].

For older adults specifically, convenient access to nonresidential destinations may play an essential facilitator of utilitarian physical activity and health, as may the presence of well-maintained sidewalks, and feelings of social cohesion/safety. Published studies in this area to date have largely relied upon self-report surveys to assess individual environment perceptions [13-17], some have employed objective environment measures [18-21], and almost all have utilized self-report physical activity. An excellent review on associations between physical activity behavior of older adults and environmental attributes was recently published [22]. To our knowledge, and indicated by this recent review, few studies have examined street-level environment characteristics in concentrated areas employing valid and reliable audit instruments and self-perceived environmental attributes compared with objective measures of physical activity specifically in older adults. Employing such rigorous methodologies will serve to greater understand the complex relationship between associations of neighborhood attributes and physical activity behavior of older adults.

Thus, this study investigated the association between objective audit and self-perceived environmental characteristics and objectively determined physical activity, hypothesizing that certain features of the environment would be associated with higher physical activity levels in older adults.

## Materials and methods

### Neighborhood selection

A total of four neighborhoods were chosen for this study. Neighborhoods were identified and data were obtained using orthophotography from the Southeastern Wisconsin Regional Planning Commission, the U.S. 2000 Census block database and shape files, and ArcView 3.2a and ArcMap 9.1 geographical informational systems (GIS) software. Two distinct neighborhood types were represented, two high-walkable (neighborhoods A and B) and two low-walkable (neighborhoods C and D). Walkability was defined upon housing density, land use, and connectivity measures [23]. Neighborhood characteristics are shown in Table 1.

**Table 1:** Neighborhood Characteristics

	High-Walkable		Low-Walkable	
**Characteristic**	**Neighborhood A**	**Neighborhood B**	**Neighborhood C**	**Neighborhood D**

Housing Density Total housing units in study area Housing density (units/acre)	1,547 14.47	1,233 11.74	511 0.99	421 0.67

Street Form Pedestrian infrastructure Street pattern Average block size (acres)	Sidewalks Merging Grids 4.45	Sidewalks Grid 5.24	No Sidewalks Curvilinear 15.09	No Sidewalks Irregular/mixed 23.09

Land Use Commercial centers Time to commercial centers (min)a	Integrated^b^ < 5	Integrated < 5	External^c^ > 15	External > 15

^a^Estimated based upon walking at 3 mph from locations within the neighborhood. ^b^Integrated refers to the combination of high residential density and residential land uses in close proximity. ^c^External refers to the strict separation of land uses.

Low-walkability neighborhoods consisted mostly of detached housing, lower density, (i.e. less than four units per acre) curvilinear street patterns, cul-de-sacs, longer block lengths, and a separation between commercial and residential areas. High-walkability neighborhoods, in contrast, had concentrations of high density (i.e., greater than four units per acre) single and multiple-family residential structures, predominantly gridlike street patterns, shorter block lengths, and a mixture of commercial integrated with residential land use.

Orthophotography were used to apply walkability criteria as defined by Saelens et al. [23]. Upon neighborhood identification, census block information was overlaid to provide a summary of neighborhood characteristics permitting an identification of targeted neighborhoods. Upon identifying two low- and two-high walkable neighborhoods, field visits were used to verify final site selection.

### Participant recruitment and study overview

A commercial marketing firm provided contact information for people residing within the selected neighborhoods of interest. An invitation mailing was sent to randomly selected households. This was then followed by phone calls (up to five attempts) to solicit study interest. Those interested were invited to the University to attend an orientation session and sign an informed consent. Following enrollment, participants came back to the University and attended two study visits separated by one-week. During visit one, participants completed a health history and demographics questionnaire, the Neighborhood Environment Walkability Scale (NEWs) survey [10], underwent measures for anthropometrics, and were asked to wear an accelerometer for a continuous 7 days. During visit two, approximately 7 days later, participants returned the accelerometer and once again repeated the NEWs survey. Data was collected between May 2005 and January 2007.

### Health history and demographics questionnaire

Participants were asked about their current, past, and family history of chronic conditions and disease. Age, sex, martial status, whether they lived alone or not, years/months living at the current address, highest education attainment, household income, and whether they had a driver's license and car were also obtained.

### Anthropometric measures

Each person underwent measures of body mass, measured to the nearest 0.01 kg, and height, measured to the nearest 0.1 cm, with minimal clothing and no shoes using a calibrated physician's scale and stadiometer (Detecto, Kansas City, MO). Body mass index (BMI) was calculated by dividing body mass (kg) by height squared (m^2^).

### Objective environmental measures

The instrument used in this investigation for objective measurement of the participant environment was the Brownson Community Audit Tool-Analytic Version (CAT-AV), as described by Hoehner and colleagues [24]. Each neighborhood in the study was broken into segments ranging from 200 ft. to 1,200 ft. Segment endpoints were determined either by the length of a defined municipal block, or at a breakpoint no further than 1,200 ft. in areas where municipal block structure was not well defined. The segments were then evaluated with respect to number and variety of land uses (e.g., residential, commercial, public institutions and recreational amenities), activity level (number and age ranges of persons visible in the segment), transportation infrastructure quality (e.g., connectivity of the street grid, parking availability, presence and width of sidewalks, transit availability) and aesthetic quality (e.g., amount of litter, noise and air pollution), as previously described [24]. Using these criteria, 983 neighborhood segments were evaluated; 316 in neighborhood A and 269 in neighborhood B (high-walkability neighborhoods), 231 in neighborhood C and 104 in neighborhood D, (low-walkability neighborhoods) and 63 in peripheral areas at the boundary of each of these communities. Each segment was given a unique identifier corresponding to a street segment on a geographic information system base map. This subsequently allowed the CAT-AV audits to be database joined to the participants' household location, leading us to analyze all segments within 656 ft. (200 m) of the participant's home location, following the street network (network buffer). Differences in buffer radii was recently shown not to impact major study outcomes between physical activity and environment attributes [25,26]. Studies have also reported perceived walkability to be approximately 400 m in regular aged adults [27]. For the purpose of the current study we elected to reduce the buffer radii to 200 m, as we believe the perceived distance to likely decrease with age.

### Perceived environmental measures

The NEWs survey [10] was self-administered to obtain perceived neighborhood attributes for each participant. Participants were instructed to consider neighborhood as the area within a 15-20 min walk from their home, and answered questions pertinent to the following subscales: a) residential density; b) proximity to nonresidential land uses; c) ease of access to nonresidential uses; d) street connectivity; e) walking/cycling facilities; f) aesthetics; g) pedestrian safety; h) crime, and i) general neighborhood satisfaction. Higher scores on the NEWs survey indicate neighborhoods which are more conducive to physical activity. The NEWs survey was administered twice, approximately 7 days apart to examine test-retest reliability in this sample of older adults.

### Physical activity measures

Physical activity was monitored by the Actigraph AM-7164 accelerometer (ActiGraph LLC, Ft. Walton Beach, Florida). The Actigraph is a lightweight (42 g) and small (5.08 × 4.06 × 1.53 cm) instrument which uses a uniaxial accelerometer powered by a lithium battery. It records accelerations from 0.05 to 2 G with frequencies of 0.25 to 2.5 Hz. The Actigraph acceleromeer has an internal time clock and extended memory, and is able to record and store the magnitude of acceleration and deceleration associated with movement. The recorded signal is then amplified and filtered, and the result is a signal that is scored as an "activity count". This count can be summed over a user-specified time interval, or epoch. Sixty-second epochs were used for this study. The monitors were attached to an elastic belt and worn at the right hip. Individuals were instructed to wear the monitor for 7 consecutive days during all waking hours, and to remove the monitor during contact with water such as bathing or swimming.

Accelerometer data considered both valid and reliable according to the following quality control procedures was used in analyses: 1) Any block of time ≥ 60 min where the accelerometer count was 0 was considered time when the monitor was not worn, 2) valid days of data were considered to be only those days during which participants wore the accelerometer for at least 600 min. Although participants were asked to wear the accelerometer for 7 consecutive days, some participants did not wear the accelerometer for the full week. Only participants who had at least 4 days of valid accelerometer data were included in this analysis.

All accelerometer data were assessed in 1 min epochs and data were coded into the following demarcations: a) total volume which was represented by average total activity counts/day; and b) time spent in light intensity physical activity which was represented by average time spent per day in activity count ranges between 50 and 759, and c) time spent in moderate to vigorous physical activity which was represented by average time spent per day in activity counts ≥ 760 counts/min [28,29].

### Statistical analysis

Descriptive statistics were calculated for demographic variables as mean ± standard deviation or as frequencies. Differences in continuous variables (i.e., age, BMI) between neighborhoods were analyzed using one-way Analysis of Variance (ANOVA). Differences in discrete variables (i.e., sex, marital status, household income, reported number of medical conditions, years/month at current address, owning a valid driving license) were calculated using chi-square tests. Total time of accelerometer determined physical activity for all intensities (i.e., total volume, light intensity, and moderate to vigorous physical activity) was calculated for all participants and by neighborhood. All analyses for accelerometer determined physical activity were controlled for wear time. Differences in physical activity between neighborhoods were assessed using a one-way ANOVA with Tukey pairwise *post hoc *testing where appropriate. For the NEWs survey, test-retest reliability statistics were calculated using Spearman correlations. Associations between physical activity and objective neighborhood characteristics were analyzed utilizing linear regression and were reported using unstandardized and standardized beta coefficients, and significance values. Similarly, associations between physical activity and perceived neighborhood characteristics (i.e., from the baseline NEWs survey) were analyzed using linear regression models. All regression models were adjusted for age, gender, BMI, years lived in current house, and driver's license/car ownership

## Results

Participant characteristics by neighborhood are presented in Table 2. The average age of this group was 64 years. Significant differences in age between neighborhoods were observed (*F*(3) = 3.634, *p *= 0.14) with *post hoc *pairwise differences seen between neighborhood A and C. The majority of this sample were women, with no differences observed in BMI, marital status, living alone or with someone else, household income, education level, the number of reported chronic conditions, or the length of time each individual had lived in their current house. Collectively this was a homogeneous group residing across 4 neighborhoods that varied by walkability.

**Table 2:** Participant characteristics by high and low walkability and neighborhood site

	**High-walkability (*n *= 86)**		**Low-walkability (*n *= 62)**		**All (N = 148)**
**Characteristic**	**Neighborhood A**	**Neighborhood B**	**Neighborhood C**	**Neighborhood D**	
Age (Yrs)	62.2 ± 8.6	62.9 ± 7.2	68.1 ± 7.9*	65.7 ± 9.1	64.3 ± 8.4
Gender (% Women)	83.3	78.9	78.8	75.8	79.7
BMI (kg/m^2^)	26.7 ± 5.7	27.0 ± 6.0	27.7 ± 5.6	28.3 ± 5.2	27.5 ± 5.6
Marital Status (% Married)	85.4	86.8	84.8	86.2	85.8
Live Alone (%)	0.04	0.05	0.03	0.0	0.03
Household Income (%)					
< $30,000/yr	18.8	13.2	15.2	13.8	15.5
> $30,000/yr	81.2	86.8	84.8	86.2	84.5
Education Attainment Level					
High School (%)	37.5	34.2	30.3	31.0	31.2
College/Advanced (%)	62.5	65.8	69.7	69.0	68.8
Chronic Conditions (#)	2.7 ± 1.7	2.9 ± 1.3	2.3 ± 1.5	2.8 ± 1.6	2.7 ± 1.5
Years in Current House (#)	26.3 ± 11.6	22.8 ± 13.1	23.4 ± 9.9	22.9 ± 10.1	23.1 ± 10.5

Note: *Different from Neighborhood A (*p *< 0.05)

### Neighborhood physical activity

Total volume of physical activity, time spent in light intensity physical activity, and time spent in moderate to vigorous physical activity for all neighborhoods are presented in Table 3. Overall, individuals residing in high-walkable neighborhoods engaged in more physical activity than those individuals residing in low-walkable neighborhoods. Across all physical activity intensities, differences were seen between neighborhood A (high-walkability) and C (low-walkabiltiy), Neighborhoods A and D (low-walkabiltiy), and between neighborhoods B (high-walkability) and C and D. No significant differences in total volume of physical activity, light intensity physical activity, or moderate to vigorous physical activity were observed between the two high-walkability neighborhoods (i.e., A and B) or between the two low-walkability neighborhoods (i.e., C and D).

**Table 3:** Participant physical activity level by high and low walkability and neighborhood site

	High-walkability (*n *= 86)		Low-walkability (*n *= 62)	
**Physical Activity**	**Neighborhood A**	**Neighborhood B**	**Neighborhood C**	**Neighborhood D**

Total Volume (activity counts/day)	326204 ± 69841	289566 ± 66712	213695 ± 76713 ^a, b^	226115 ± 72487 ^a, b^

Light Intensity PA (min/day)	290.8 ± 79.4	287.6 ± 69.6	243.7 ± 46.2 ^a, b^	246.4 ± 58.9 ^a, b^

Moderate to vigorous PA (min/day)	26.5 ± 10.0	29.6 ± 12.1	16.3 ± 5.3 ^a, b^	18.8 ± 6.7 ^a, b^

*Note: PA *Physical activity. ^a^Significantly different from Neighborhood A, *p *< 0.05. ^b^Significantly different from Neighborhood B, *p *< 0.05. Activity counts/day calculated by 60 second epochs. Light intensity PA represents minutes per day with accelerometer counts per minute between 50-759. Moderate to vigorous physical activity represents minutes per day with accelerometer counts per minute of ≥ 760.

### Association between measured environment and objective physical activity

Results from the linear regression model based on objective neighborhood characteristics showed the model for total volume of physical activity was significant (*F*(19, 129) = 4.80, *p *< .001; R^2 ^= .373) with a significant effect from non-residential destinations (beta = 854, *p *= .005) and crime (beta = 4784.5, *p *= .031). No other independent variables in the model were significant. The model for time spent in light intensity physical activity was also significant (*F*(16, 129) = 4.34, *p *< .001; R^2 ^= .350). Both non-residential destinations (beta = -4.2, *p *= .033) and street safety score (beta = 26.81, *p *= .006) were significant predictors for time spent in light intensity physical activity. The model for time spent in moderate to vigorous physical activity was significant (*F*(16, 129) = 9.38, *p *< .001; R^2 ^= .538), however only non-residential destinations was a significant predictor (beta = .11, *p *< .001) of time spent in moderate to vigorous physical activity. No other independent variables had a significant effect on time spent in moderate to vigorous physical activity in this model. Table 4 shows the unstandardized and standardized beta coefficients, and corresponding significance value for each environmental audit measure characteristic for all physical activity intensities.

**Table 4:** Unstandardized and standardized Beta coefficients from linear regression model for objective evaluation of neighborhoods by physical activity (N = 148)

	Total Volume			Light Intensity PA			Moderate to vigorous PA		
**Environmental Measure**	**β**	**B**	**P value**	**β**	**B**	**P value**	**β**	**B**	**P value**

**Land Use **Nonresidential destinations^a^	**853.71**	**.35**	**.005**	**-4.15**	**-.27**	**.033**	**.11**	**.48**	**< .001**

**Recreational Facilities **Parks^b^	-8900.96	-.25	.114	-4.87	-.22	.174	-.04	-.01	.925

Any Park^c^	-21855.26	-.10	.286	-11.25	-.08	.388	-.51	-.02	.766

Count of Facilities^d^	4902.15	.20	.260	-.73	-.05	.791	-.13	-.05	.723

**Transportation **Sidewalk Present^e^	55984.07	.23	.240	5.80	.04	.848	3.75	.16	.348

Bike lanes Present^f^	31866.68	.12	.157	-5.66	-.03	.692	-1.38	-.05	.472

Public Transit^g ^	-19410.82	-.04	.715	-27.04	-0.9	.425	-7.12	-.14	.110

Street Safety^h^	20896.16	.28	.164	**26.81**	**5.8**	**.006**	.53	.07	.673

**Aesthetics** Attractive Features^i^	-18507.81	-.05	.568	-30.57	-.14	.140	-2.15	-.06	.431

Comfort amenities^j^	18772.54	.02	.846	-7.24	-.01	.906	5.73	.07	.480

Litter^k ^	244.76	.01	.922	-1.83	-.139	.249	-.09	-.04	.681

**Social **Crime^l^	**4784.46**	**.18**	**.031**	.19	.01	.892	-.04	-.02	.822

Engagement in Activity^m^	602.88	.21	.236	.44	.25	.173	.05	.17	.277

*Note: PA *Physical Activity. Total volume of PA = average accelerometer counts per day. Light intensity PA represents minutes per day with accelerometer counts per minute between 50-759. Moderate to vigorous physical activity represents minutes per day with accelerometer counts per minute of ≥ 760.

Models adjusted for age, gender, body mass index, years lived in current house, and driver's license/car ownership. The following descriptions of audit measures are all pertinent to within a 200 m radii of the individuals home: ^a^Sum of nonresidential destination including those related to retail, service, entertainment, schools, employment, and civic organization; ^b^Sum of number of parks with facilities including walking trails, sports fields/courts or playgrounds; ^c^Presence of at least one park, trail, indoor fitness facility; ^d^Sum of the number of recreational facilities including parks, trails, sports fields/courts, outdoor pools and indoor recreational facilities; ^e^Percent of street segments with even sidewalks; ^f^Presence of a bike lane; ^g^Percent of street segments with a bus or transit stop; ^h^Average street safety score calculated by segment by summing audits related to number of traffic lanes, connectivity, street design to reduce speed, traffic calming devices, aggressive drivers (reverse coded), crossing aids and street lighting; ^i^Percent of street segments with attractive features such as vegetation, building variety and architectural design; ^j^Percent of segments with comfort features such as tree shade and benches; ^k^Percent of segments with little garbage or litter; ^l^Sum of weighted response assessing presence of neighborhood crime or crime watch signage; ^m^Sum of weighted response assessing number of individuals engaging in active behaviors.

### Association between perceived environment and objective physical activity

When examining test repeatability (i.e., test-retest) of the NEWs survey [10], results showed moderate to strong significant Spearman correlation coefficients in all subcategories (*p *< .001). Rho values ranged from .63 for the d subscale to .88 for the b subscale. Subscales a (.82), b (.88), c (.75), e (.83), f (.79) and i (.85) all had high rho values (> .70) indicating good test-retest repeatability in this sample of older adults.

The regression model based on the NEWs survey resulted in a significant model for total volume of physical activity (*F*(12, 130) = 2.67, *p *= .003; R^2 ^= .198), however no subscale predictors were significant with the singular exception of neighborhood satisfaction; beta = -48166.1, *p *= .028. The model for time spent in light intensity physical activity was significant (*F*(12, 130) = 3.18, *p *< .001; R^2 ^= .227), with proximity to non-residential destinations as a significant predictor (beta = 18.62, *p *= .046). For time spent in moderate to vigorous physical activity, the model was significant (*F*(12, 130) = 5.09, *p *< .001; R^2 ^= .320). Proximity to non-residential destinations neighborhood satisfaction were positive, significant predictors (beta = 2.89, *p *= .031 and beta = 5.79, *p *= .003, respectively). Table 5 shows the unstandardized and standardized beta coefficients, and corresponding significance value for each NEWs subscale for all physical activity intensities.

**Table 5:** Unstandardized and standardized Beta coefficients from linear regression model for subjective evaluation of neighborhoods (Neighborhood Environment Walkability Scale; NEWs) by physical activity (N = 148)

	Total Volume			Light Intensity PA			Moderate to vigorous PA		
**Environmental Measure**	**β**	**B**	**P value**	**β**	**B**	**P value**	**β**	**B**	**P value**

Residential density^a^	224.09	.13	.163	-.10	-.10	.287	.000	.00	.973

Proximity to nonresidential land uses^b^	13795.29	.12	.364	**18.62**	**.27**	**.046**	**2.89**	**.27**	**.031**

Ease of access to nonresidential uses^c^	6728.54	.04	.750	4.26	.05	.742	.53	.04	.775

Street connectivity^d^	2061.46	.01	.910	2.81	.03	.801	.36	.02	.821

Walking/cycling facilities^e^	16643.18	.16	.251	9.07	.14	.306	1.67	.17	.189

Aesthetics^f^	2979.34	.02	.906	3.94	.03	.799	1.98	.10	.374

Pedestrian Safety^g^	33830.20	.17	.123	17.82	.19	.109	1.72	.09	.370

Safety from Crime^h^	30369.78	.15	.241	18.98	.15	.231	2.11	.17	.181

Neighborhood Satisfaction^i^	**48166.11**	**.30**	**.028**	-23.37	-.23	.079	**5.79**	**.38**	**.003**

*Note*: *PA *Physical activity. Total volume of PA = average accelerometer counts per day. Light intensity PA represents minutes per day with accelerometer counts per minute between 50-759. Moderate to vigorous physical activity represents minutes per day with accelerometer counts per minute of ≥ 760.

Models adjusted for age, gender, body mass index, years lived in current house, and driver's license/car ownership. The following descriptions of perceived environmental measures stem from the scoring procedures used for the Neighborhood Environment Walkability Scale (NEWs). ^a^Residential density, types of residences in your neighborhood, detached single family residences to multi story apartments/condos; ^b^Proximity to nonresidential land uses by walking, stores facilities and other things in your neighborhood 1-5 min, 6-10 min, 11-20 min, 21-30, and 31+ min away; ^c^Ease of access to nonresidential uses, access to other services within a 10-15 min walk from your home; ^d^Street connectivity, cul-de-scas, walkways, intersection length, presence of four-way intersections, varying routes; ^e^Walking/cycling facilities, presence of sidewalks, level of maintenance, ease of access, separated from traffic; ^f^Aesthetics, neighborhood surroundings, presence of trees, tree shade, interesting visuals, attractiveness, lack of litter; ^g^Pedestrian safety, safety from traffic, level, closeness, speed of traffic, speed control elements, degree of crosswalks, transport pollutants; ^h^Safety from crime, level of lighting, presence of others, crime rating; ^i^Neighborhood satisfaction.

## Discussion

The current study extends the literature linking neighborhood attributes to physical activity by examining both perceived and measured environmental characteristics in association with measured physical activity behavior specific to older adults at the neighborhood (macro) and resident level (micro). This study, by design, specifically recruited from *a priori *identified neighborhoods that elicited environmental attributes deemed to be either more- or lesswalkable. Physical activity levels were then compared at the macro neighborhood level, and then also at the individual resident micro level examining in depth measured neighborhood characteristics at the 200 m buffer radii level, and also those self-perceived by the individual. The principal findings from this study showed those residing in high-walkable neighborhoods were more physically active than those residing in low-walkable neighborhoods. Collectively, at the individual resident level measured environmental attributes jointly accounted for 38% of total activity, 35% of time spent in light intensity activity, and 54% of time spent in moderate to vigorous physical activity, after accounting for age, gender, body mass index, years lived in residence, and driver's license/car ownership. In general these results were stronger than those observed from self- perceived environmental attributes, jointly accounting for 20%, 23% and 32% of total, time spent in light, and time spent in moderate to vigorous physical activity, respectively. Proximity to non-residential destinations was a common association with physical activity at the individual resident level, regardless of whether this was measured objectively, or self-perceived by the individual. Other individual resident level measured and perceived environmental attributes yielded a lack of concordance with objectively measured physical activity levels.

Older adults who lived in neighborhoods that were characterized as being high-walkable on average engaged in approximately 45 min more of light intensity physical activity per day, and approximately 11 min more of moderate to vigorous physical activity per day. Older adults who had, and who perceived to have, destinations such as retail, services and entertainment close by were likely to be more physically activity. Such findings are consistent with other studies. Michael and colleagues in their study of 105 older adults drawn from 10 neighborhoods as part of the larger SHAPE trial [30] revealed that the presence of walking destinations was related to higher self-reported walking behavior [20]. King et al. in a group of 158 older women reported amenities within walking distance to individual residences to be associated with pedometer determined walking behavior [31]. Nagel and coauthors further extended these relations and found that there are stronger relationships for linkages between physical activity and commercial establishments for those who already report some degree of walking behavior [26]. Collectively, past studies, and current results demonstrate that proximity to non-residential destinations is important for older adult physical activity behavior.

The current study also found that measured street safety scores, which encompasses the number of traffic lanes, street design to reduce speed and other traffic calming devices, along with an overall connectivity measure was associated with total volume of physical activity (beta = 26.8, *p *= -.006). We did not find self-perceived street connectivity and pedestrian safety to be associated with physical activity. Current literature findings are generally unequivocal in this area, with Li and colleagues noting walking levels to be higher in areas with greater street connectivity [19], and Michael and co-workers finding an overall lack of association with street level characteristics [20]. Often, features of street connectivity are consonant with other aspects of land-use mix, and proximity to nonresidential destinations. It may be that in collection, street level characteristics are reduced in importance, or become overcome by other attributes in their predicative relationship to, or associations with, physical activity in older adults. A lack of concordance between perceived and measured attributes is likely indicative that other interesting interactions are present that warrant further investigation in an attempt to fully discern contributions to physical activity behavior.

The social environment is important and is likely to influence physical activity in older adults.. Current results support measured social environmental attributes pertinent to neighborhood crime or crime watch signage being associated with total volume of physical activity (beta = 4784.5, *p *= -.031). Other studies have indicated that factors such as social cohesion [32] are important in explaining senior activity levels, and our own work has highlighted a likelihood to be more active if others are also physically active, by way of providing social support and an increased sense of safety [33]. Parallel to the aforementioned ideals, was the current study findings that overall perceived neighborhood satisfaction was related to both total activity (beta = 48166.11, *p *= -.028) and time spent in moderate to vigorous physical activity (beta = 5.79, *p *= -.003). This self-perception in concurrence with other land-use findings supports the notion that the environment is an important facilitator to older adult physical activity behavior. These findings, in combination with other literature findings pertinent to older adults are consistent with studies from the general adult population that highlight the linkage between built and social neighborhood attributes and physical activity engagement [34].

The current study is not without limitation. By design, causality cannot be inferred. Although utilizing objective measurement of physical activity behavior, hereby reducing the limitations of self-report activity, is a strength to study design, domain specific physical activity cannot be evaluated. By definition, the built and social environment is more likely to impact transport and utilitarian physical activity. Such domain specific features of physical activity are likely to be consumed by a total activity metric, and results linking environment to physical activity potentially weakened as a result. Future studies investigating the measured and perceived environmental attributes of domain specific physical activity would enhance this understanding. By employing technologies such as global positioning systems, investigative teams would be well served to show overall location and domain specificity in conjunction with objective physical activity outcomes.

## Conclusions

Results extend the current literature, and support that older adult residents who live in high-walkable neighborhoods, who have easy and close access to nonresidential destinations, have lower social dysfunction pertinent to crime, and generally perceive the neighborhood to a higher overall satisfaction are likely to engage in higher levels of physical activity behavior. Growing evidence in this area strongly suggest that the neighborhoods in which older residents live are fundamentally important to physical activity levels, and ultimately health. Policies and practices to create activity friendly senior environments are essential to alleviate health ailments associated with current aging and aging and inactivity societal trends.

## Competing interests

The authors declare that they have no competing interests.

## Authors' contributions

SJS, MJG and RI conceptualized and conducted the study. AMS assisted with study design, and participated in study coordination. TLH, EKL and CJD assisted with data management and analysis. All authors contributed to manuscript writing, modified and approved the final version.
